# Preclinical Efficacy of a Hemostatic Agent in Overcoming Dual Antiplatelet Therapy

**DOI:** 10.1016/j.jacbts.2025.101356

**Published:** 2025-08-15

**Authors:** Evgeni Efimenko, Hairu Zhao, Keith Moskowitz, Conrad Smith, Robert Pyo, Thomas G. Diacovo

**Affiliations:** a Division of Neonatology, Stony Brook University, Stony Brook, New York, USA; b Department of Pediatrics, Stony Brook University, Stony Brook, New York, USA; c Department of Pediatrics, University of Pittsburgh, Pittsburgh, Pennsylvania, USA; d Cellphire Therapeutics, Rockville, Maryland, USA; e Division of Cardiology, Stony Brook University, Stony Brook, New York, USA; f Department of Medicine, University of Pittsburgh, Pittsburgh, Pennsylvania, USA; g Department of Medicine, Stony Brook University, Stony Brook, New York, USA

**Keywords:** coronary stent, DAPT, hemostatic agent, microfluidics, thrombin

## Abstract

This preclinical evaluation of a prohemostatic agent involved patients who received aspirin and clopidogrel before coronary artery stenting, the use of a humanized animal model to assess the hemostatic properties of patient platelets, as well as microfluidic assays to measure platelet reactivity. We demonstrate that our investigational product can bypass the effects of dual antiplatelet therapy (DAPT) by generating thrombin at sites of vascular injury, thereby restoring the hemostatic properties of patient platelets. Importantly, its effects could be reversed upon administration of a thrombin inhibitor. Thus, this product offers a titratable and reversible strategy for the management of defective hemostasis associated with DAPT.

## Highlights

• FPH can form an effective, nonocclusive hemostatic plug at sites of vascular injury.
• FPH can restore the hemostatic capacity of DAPT exposed platelets in a VWF- and dose-dependent manner.
• FPH is agnostic to P2Y_12_ antagonism and can overcoming DAPT inhibition through thrombin generation.

## Visual Abstract

**Figure:**
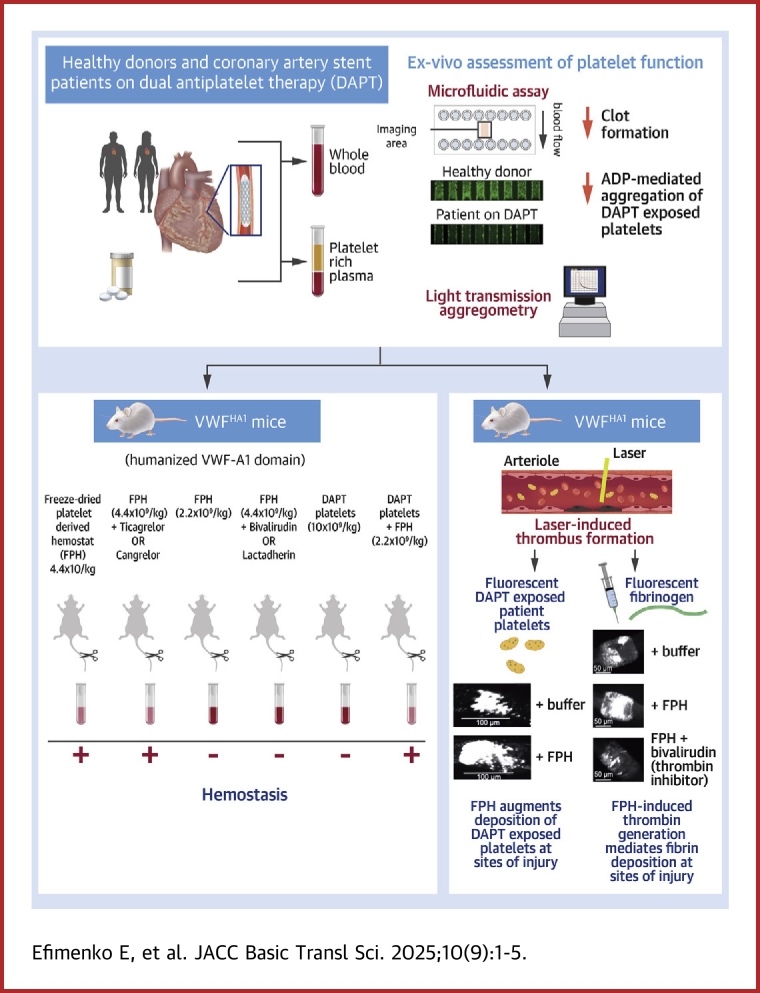

Platelets play an important role in primary hemostasis due to their ability to rapidly adhere to and subsequent form a primary hemostatic plug on exposed collagen at sites of arterial injury. The adenosine diphosphate (ADP) receptor P2Y_12_ is an essential part of this process as its activation potentiates the release of platelet dense granule constituents that contribute to aggregation, promote procoagulant activity through membrane exposure of phosphatidylserine (PS), and enhance the efficiency of platelet activation by other agonists such as thrombin.^1^^,^^2^ The importance of this pathway is underscored by the congenital bleeding diathesis in humans associated with molecular defects in the P2Y_12_ receptor.^3^

As the P2Y_12_ receptor plays a central role in platelet aggregation, it remains a primary pharmacologic target for the prevention of myocardial infarction and stroke in patients with significant cardiovascular disease.^4^, ^5^, ^6^ In fact, the clinical usage of P2Y_12_ receptor antagonists (eg, clopidogrel, ticagrelor, cangrelor, and prasugrel), in combination with aspirin, has significantly decreased the risk of thrombotic complications associated with atherosclerotic cardiovascular diseases.^7^ Thus, it is considered a central strategy in the prevention of coronary artery thrombosis after stent placement.^8^^,^^9^

Despite the proven efficacy, patients on dual antiplatelet therapy (DAPT) are at increased risk for life-threatening bleeds that contribute to mortality after coronary artery stent placement.^10^^,^^11^ This includes individuals requiring coronary artery graft bypass, vascular and abdominal surgeries, and can also manifest as spontaneous gastrointestinal hemorrhage.^12^^,^^13^ This is further compounded by comorbidities that include advanced age, stroke, liver dysfunction, trauma, cancer, and thrombocytopenia.^14^ Several strategies have been considered to reduce the significant health and economic burdens for both patients and institutions including de-escalation of DAPT intensity and duration, as well as the more judicious use of platelet transfusion in the prevention and treatment of bleeding.^15^, ^16^, ^17^, ^18^ In the latter case, only modest efficiency has been achieved in the recovery of hemostatic function due in part to excess free antiplatelet agents (APA) or their active metabolites in blood inhibiting their respective targets on transfused platelets.^19^, ^20^, ^21^ Thus, there remains an unmet need to develop an agent or agents resistant to the effects of APAs to achieve rapid hemostasis in patients who are at risk for significant hemorrhage due to trauma, urgent cardiac and noncardiac surgical procedures, or an acute gastrointestinal event.

Based on the previously described characteristics of a freeze-dried, platelet-derived hemostatic agent (FPH) (previously known as thrombosomes) that includes universal compatibility and an acceptable safety profile in humans, we hypothesized that this agent may prove advantageous as a readily available therapy to overcome the direct effects of DAPT in cardiovascular patients with life-threatening bleeds.^22^, ^23^, ^24^, ^25^ This is supported by the ability to directly interact at sites of vascular injury despite the absence of outside-in/inside-out signaling and mitochondrial activity.^26^ However, it remains unclear whether FPH would be efficacious in conditions where P2Y_12_ signaling pathway is disrupted and if it can prevent/ameliorate bleeding by augmenting endogenous circulating platelets to form stable hemostatic clots. To this end, we tested the ability and explored the mechanism(s) by which this platelet derived product supports hemostasis directly and indirectly using a humanized Von Willebrand factor (VWF) mouse in which platelets from cardiovascular patients on DAPT were infused.

## Methods

### Freeze-Dried, Platelet-Derived Hemostat

FPH were manufactured and reconstituted as previously described.^23^ In brief, 10 leukocyte-reduced Type O platelet apheresis units collected and stored under blood bank conditions were pooled, diluted into a proprietary lyophilization buffer containing trehalose, polysucrose, and ethanol, then concentrated and washed by ultrafiltration and diafiltration. Glass vials, with a volume capacity of 100 mL or 200 mL, were then filled aseptically with 10 mL or 30 mL of the suspension, respectively. The platelet-derived product was lyophilized, sealed under vacuum, and then heat treated at 80 °C for 24 hours. The consistency of the product upon reconstitution with sterile water was confirmed by performing a particle count (2.09 ± 0.07 × 10^9^/mL; mean ± SEM), measuring size (1-5 μm ± 1.6 μm; mean ± SEM) and determining purity (98.7% ± 0.18% positive for GPIIb IIIa; mean ± SEM). In addition, each lot of FPH must generate a similar level of thrombin as previously agreed upon with the Food and Drug Administration.^26^ Reconstituted product was also cultured to detect any aerobic or anaerobic bacteria and tested for endotoxin before release.

### Reagents

Cangrelor tetrasodium salt (Sigma-Aldrich) and Ticagrelor (R&D Systems) were used at final concentrations of 0.1 μmol/L and 2 μmol/L for in vitro studies. For in vivo analyses, cangrelor, tirofiban, and bivalirudin were obtained from the hospital research pharmacy and reconstituted per the manufacturers’ recommendations; ticagrelor was diluted in methylcellulose/Tween 80. Aspirin (MilliporeSigma) and clopidogrel (Sanofi) were dissolved in distilled water.^27^ ADP, type I equine collagen, luciferin-luciferase reagent (Chrono-Lume), and ATP standard were obtained from Chrono-Log Corporation; the PAR-1 hexapeptide agonist TRAP-6 was purchased from BACHEM. The thrombin inhibitor H-D-Phe-Pro-Arg-chloromethylketone (PPACK), Alexa Fluor 488–conjugated mouse anti-human CD61, and Alexa Fluor 647–conjugated human fibrinogen were obtained from Bio-Rad and Thermo Fisher Scientific, respectively. The mouse anti-human GPIb alpha monoclonal antibody (mAb) 6D1 was a generous gift of Barry Coller (Rockefeller University, New York, New York).

### Mice

Human VWF A1 domain (VWF^HA1^) mutant animals and wild-type (WT) littermates, both on a NOD/SCID background, were generated as previously described.^28^ Von Willebrand factor knockout (VWF^KO^) mice were purchased Jackson Laboratory. All procedures were approved by the Institutional Animal Care and Use Committees at Stony Brook University (SBU).

### Blood Collection

Blood was obtained via venipuncture from healthy adult volunteers and patients on DAPT undergoing coronary artery stenting by drawing into a BD syringe containing either 3.8% trisodium citrate or 100 μmol/L PPACK as anticoagulant, the latter used in microfluidic (MF) studies.^29^ Participants were enrolled at the University of Pittsburgh Medical Center (UPMC) and SBU between January 2023 and October 2023. DAPT was administered per care team and institution guidelines. This study was approved by the UPMC and SBU institutional review boards/ethic committees and conducted in accordance with the Declaration of Helsinki and ICH Good Clinical Practice. Written informed consent was obtained from each participant before study-related activities.

### Platelet Aggregation and MF Analyses of Thrombus Formation

To standardize light transmission aggregometry (LTA) studies, platelets were isolated by centrifugation of citrated blood samples and then suspended to a final concentration of 250,000/μl in assay buffer (145 mmol/L NaCl, 10 mmol/L HEPES, 0.5 mmol/L Na_2_HPO_4_, 5 mmol/L KCl, 2 mmol/L MgCl_2_, 1 mmol/L CaCl_2_, 0.1% glucose, pH 7.4) as previously described.^30^ Human fibrinogen (final concentration 200 μg/mL) was added to suspensions just before platelet activation. Isolated patient or healthy donor platelets were stimulated with 20 μmol/L ADP or 10 μmol/L TRAP-6 final concentrations. To evaluate the ability of FPH to absorb P2Y_12_ receptor antagonists from plasma, platelets from healthy donors were suspended in autologous platelet-poor plasma (PPP) that was first treated with either cangrelor or ticagrelor, incubated with FPH (15 minutes) and subsequently depleted of FPH by centrifugation before performing LTA (20 μmol/L ADP). Aggregation was assessed with a Chronolog Lumi-Aggregometer (model 540 VS, Chronolog) and permitted to proceed for 6 minutes after the addition of agonist. Aggregation results are reported as maximum percent change in light transmittance from baseline with platelet buffer used as a reference.

For MF assays, 2 approaches were used to evaluate thrombus growth in real time during infusion of PPACK-treated whole blood. The first involved an 8-channel device fabricated in polydimethylsiloxane (PDMS) that was vacuum attached to a type 1 equine collagen–coated glass slide and utilized an automated epifluorescence microscopy system (Leica DMi8, PL FLUOTAR 10×/0.3 objective) and syringe pump (Harvard Apparatus).^29^ The second involved a self-contained, microfluidic device (FloBio) that utilizes an 8-sample well, injection-molded microfluidic chip with surface-immobilized collagen, and contains a self-analyzer including a vacuum pump, LEDs, optics, and automated control functionality to measure accumulation of Alexa Fluor 488–labeled platelets in whole blood under flow conditions.^31^

### Ex Vivo Thrombin Generation and PS Blockade

A thrombin generation assay was developed on a CLARIOstarPlus plate reader (BMG Labtech) to evaluate the impact of blocking exposed PS on the surface of FPH.^32^ To this end, the product was incubated with recombinant bovine lactadherin (Prolytix) at concentrations of 0, 25, 50, 100, and 200 μg/mL for 30 minutes at room temperature. FPH was then diluted 1:32, 1:64, and 1:128 in buffer and added at 10 μL per well in a 96-well plate. Each well received 70 μL of Octaplas plasma, 15 μL of tissue factor containing PRP-reagent (Diagnotica Stago), and 5 μL of 60 mmol/L Gly-Pro-Arg-Pro (GPRP, BACHEM). The raw fluorescence intensity of a fluorogenic thrombin substrate, FluCa (Stago), added at 40 μL per well, was measured over time. The first derivative of that signal was calculated to give the maximum thrombin generation rate in raw fluorescence intensity/second. A standard curve was plotted based on maximal thrombin generation values of thrombin standards (Sigma Aldrich).

### Blood Coagulation Test

Prothrombin time (PT) and activated partial thromboplastin time (aPTT) were measured using clotting assays with reagents used for human clotting factor determination in an automated coagulation analyzer (ACL TOP 750^CTS^, Werfen). Blood was collected by cardiac puncture using a syringe containing 3.8% trisodium citrate 30 minutes after mice received an intravenous bolus of either buffer control or FPH (4.4 × 10^9^ particles/kg of body weight).

### In Vivo Thrombus Formation

Administration of anesthesia, insertion of vascular catheters, fluorescent labeling of platelets, surgical preparation of the cremaster muscle has been previously described.^28^^,^^30^ A pulsed nitrogen dye laser was used to induce arteriole injury in 10- to 12-week-old animals. Interactions of CFDA-SE–labeled FPH, calcein-labeled human, or rhodamine 6G–labeled mouse platelets with the injured vessel wall were visualized by intravital fluorescence microscopy using the Andor Dragonfly high-speed confocal platform with 488-nm, 561-nm and 637-nm laser lines, an automated Leica DM6 upright microscope containing a 25×/0.95 water immersion objective, and MicroPoint ablation system (Andor Technology-Oxford Instruments). Experiments were performed in accordance with the guidelines set forth by Institutional Animal Care and Use Committee at UPMC and SBU.

To assess in vivo fibrin production (surrogate for thrombin activity), VWF^HA1^ mice received an intravenous injection of Alexa Fluor 647–conjugated human fibrinogen (1 mg/mL). FPH alone or in combination with the direct thrombin bivalirudin (4 μg/g body weight) was subsequently infused and vascular damage initiated in arterioles of similar size. Z-axis image stacks (1-μm sequential sections; total 50 μm) were taken at 1, 3, and 5 minutes after injury. Data sets were flattened along the z-axis as maximum intensity projections to enable determination of the total fibrin area using Leica Aivia AI Image Analysis Software.

### Tail Bleeding Times

Bleeding times were measured in anesthetized 8-week-old VWF^HA1^ mice after amputating 1 cm of tail tip and then placing tail in a physiological saline solution (37 °C) for durations ranging from 10 to 20 minutes.^28^ Animals received human platelets, FPH, cangrelor and/or bivalirudin by intravenous route 1 minutes before tail amputation. Ticagrelor was diluted in methylcellulose/Tween 80 and administered by oral gavage (30 mg/kg). Aspirin and clopidogrel were dissolved in distilled water and administered at 5 mg/kg via oral gavage either 4 h or 24 h and 4 h before experiments, respectively.^27^ In addition, animals possessing the native (WT) murine VWF-A1 domain received the identical concentration of antiplatelet agents in the absence of FPH to demonstrate that each drug or combination of drugs disrupted the ability of murine platelets to support effective hemostasis.

To evaluate the role of exposed PS in contributing to thrombin production and hence hemostasis, FPH (4.4 × 10^9^ particles/kg of body weight) was preincubated the lactadherin at 200 μg/mL for 30 minutes at RT. Subsequently, 150 μL of FPH or buffer control was injected intravenously into VWF^HA1^ mice and 1 cm of tail tip amputated; bleeding was evaluated as described in the preceding text.

### Statistical Analysis

Baseline characteristics are presented as the median with 25th-75th percentiles (Q1-Q3) or count (percentage). Results are presented as the mean ± SD with 95% CI or by plotting the data points with the mean and 10th-90th percentiles. Statistical analyses were performed with GraphPad Prism 10.3.0 (GraphPad Software). For non-normally distributed (determined by D'Agostino-Pearson omnibus normality test), unequal variance data, or those with n < 6, Mann-Whitney nonparametric *U* test was used. For multiple comparisons analysis, 1-way analysis of variance with post hoc Tukey Honestly Significant Difference was used to compare differences. Kaplan-Meier survival plots were analyzed using the log-rank test to determine effects on hemostasis as assessed by tail bleeding times.^33^ Detailed methods for assessing statistical significance are presented in the corresponding figure legends. The exact group size (n) for each experiment is provided in the figure legends, and n refers to biological replicates. The precise *P* value for significance is indicated in each figure. A *P* value <0.05 was considered statistically significant.

## Results

### Patient Characteristics

Twenty-seven patients undergoing coronary artery stent placement were approached and enrolled in this study from August 2022 to October 2023. Of these, 22 were men and 5 were women with a mean patient age of 71 years (Q1-Q3: 59-89 years). Twenty (74.1%) were on aspirin (81 mg) and 24 (88.9%) on clopidogrel (75 mg) before the intervention. Twenty (74.1%) received an additional loading dose of aspirin (total dose 325 mg), and 11 (40.7%) clopidogrel (total dose 300 mg) per institutional guidelines. Mean platelet count was 175 × 10^3^/μL (Q1-Q3: 75-381 × 10^3^/μL). Table 1 list patient demographics and medical history.

**Table 1:** Baseline Characteristics and Study Treatments (N = 27)

Age, y	71 (59-89)
Sex	
Men	22 (81.5)
Women	5 (18.5)
Race/ethnicity	
White	26 (93.3)
Black	1 (3.7)
Other	0
Weight, kg	84 (56-127)
Body mass index^a^	29 (22.1-39.2)
Medical history	
Hypertension	23 (85.2)
Hyperlipidemia	27 (100)
Diabetes mellitus	5 (18.5)
AF	6 (22.2)
Cirrhosis	2 (7.4)
CRF	7 (25.9)
Stroke/TIA	0
Prior PCI	20 (74.1)
Prior CABG surgery	4 (14.8)
Antiplatelet medications	
Daily clopidogrel, 75 mg	24 (88.9)
Daily aspirin, 81 mg	20 (74.1)
Load clopidogrel, total 300 mg	11 (40.7)
Load aspirin, total 325 mg	20 (74.1)
Platelet count × 10^3^/μL	175 (75-381)

Values are median (Q1-Q3) or n (%).

AF = atrial fibrillation; CABG = coronary artery bypass graft; CRF = chronic renal failure; PCI = percutaneous coronary intervention; TIA = transient ischemic attack.

a Body mass index is calculated as weight in kilograms divided by height in meters squared.

### Assessment of DAPT-Exposed Platelet Reactivity

**Figure 1:**
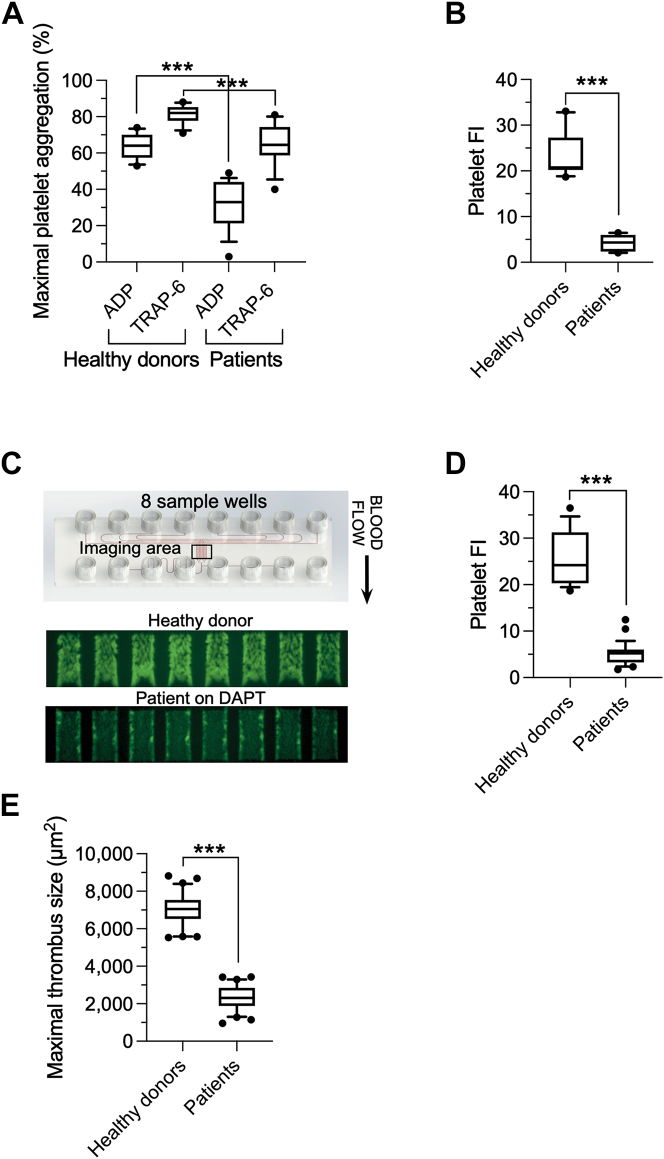
In Vitro Assessment of Platelet Reactivity by Aggregometry and MF (A) Percent maximal aggregation of platelets from patients on dual antiplatelet therapy (DAPT) (n = 27) in response to adenosine diphosphate (ADP) (20 μmol/L) and TRAP-6 (10 μmol/L). Results are compared with healthy donors (n = 15). (B) Mean fluorescence intensity (FI) (n = 8 clotting events per individual) of accumulated platelets from whole blood of healthy donors vs patients on DAPT using a PDMS (polydimethylsiloxane) device attached to a type I equine collagen–coated glass slide. Digital imagines were captured using an automated fluorescent microscope system (*t* = 900 seconds). (C) Photomicrograph of a fabricated collagen-coated chip and deposition of platelets labeled with Alexa Fluor 488–conjugated mouse anti-human CD61 from healthy donors or DAPT-treated patients (*t* = 900 seconds). (D) Mean platelet FI (n = 8 clotting events per individual) of accumulated platelets from WB of the same individuals from using collagen-coated chips and an integrated microfluidic (MF) device. (E) Maximal thrombus area (μm^2^) occupied by calcein-labeled platelets from healthy donors vs patients (n = 10 mice per group, 10 individuals for each of the 2 groups, 3 arteriole injuries per mouse). The central box represents the values between the 10th and 90th percentiles, and the middle line is the mean. Statistical significance was determined using Mann-Whitney *U* test. ∗∗∗*P* < 0.001 relative to healthy donors; ∗∗∗*P* < 0.001 ADP relative to TRAP-6 for patients on DAPT.

The extent of inhibition in reactivity of DAPT-exposed platelets from patients undergoing coronary artery stenting was determined by LTA and in 2 MF assays, the latter using PPACK-treated whole blood. ADP-mediated platelet aggregation was significantly reduced (*P <* 0.001) in all patients compared with healthy donors with a mean ± SD maximal platelet aggregation detected by LTA of 32% ± 13.4% (95% CI: 25.3%-38.7%) vs 63.8% ± 6.9% (95% CI: 59.4%-68.2%), respectively (Figure 1A). However, there remained a relatively robust response to the PAR-1 receptor agonist TRAP-6, albeit not to the extent observed for healthy donors (64.4% ± 11.1% [95% CI: 59%-70%] vs 81.3% ± 4.9% [95% CI: 78.2%-84.4%], respectively).

To assess platelet reactivity under conditions that better reflect the in vivo milieu present at sites of vascular injury, we first used a PDMS device/microscopy-based MF system with proven ability to detect the effects of acetylsalicylic acid (ASA) and P2Y_12_ inhibitors on platelet function in response to surface-immobilized type 1 collagen as substrate.^29^ Consistent with LTA results, a significant reduction in clot formation was observed for PPACK-treated whole blood from patients on DAPT as compared with healthy donors yielding a mean ± SD fluorescence intensity (FI) of 4.2 ± 1.8 (95% CI: 3-5.5) vs 23.3 ± 5 (95% CI: 19.7 to 26.9), respectively (Figure 1B). Of note, nearly identical results were observed with a newly developed, self-contained MF device that utilizes manufactured type 1 collagen–coated chips: mean FI of 5 ± 2.4 (95% CI: 4.1-6) for patients vs 25.8 ± 6.1 (95% CI: 22.4-29.8) for healthy donors (Figures 1C and 1D). Importantly, the hyporesponsiveness of platelets from patients on DAPT was confirmed in vivo as evidenced by the formation of significantly smaller thrombi in injured arterioles of VWF^HA1^ mice as compared with healthy donors: mean area of 2,241 μm^2^ ± 616 μm^2^ (95% CI: 2,010-2,471 μm^2^) vs 7,347 ± 774.1 μm^2^ (95% CI: 7,058-7,602 μm^2^), respectively (Figure 1E). Overall, the combination of ASA and clopidogrel resulted in a 69% vs 80% reduction in the ability of platelets to form clots as detected in vivo vs MF assays, respectively.

### Hemostatic and Thrombotic Properties of FPH

**Figure 2:**
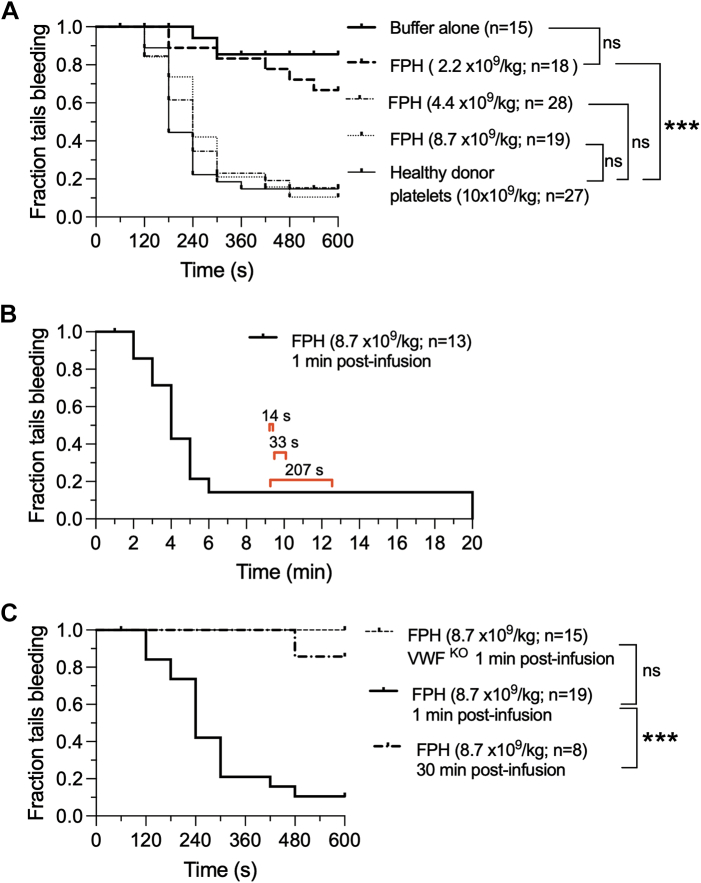
FPH Corrects the Hemostatic Defect in VWF^HA1^ Mice but Not VWF-Deficient Animals Fraction of tails bleeding at (A) 10 minutes vs (B) 20 minutes for human Von Willebrand factor A1 domain (VWF^HA1^) mice immediately after administration of freeze-dried, platelet-derived hemostatic agent (FPH) at the indicated dosing concentrations. In the former, results are compared with platelets from healthy human donors. In the latter, brackets indicate the duration of the bleeding event observed for 3 animals before re-establishing hemostasis. (C) Fraction of tails bleeding for VWF^HA1^ vs Von Willebrand factor knockout (VWF^KO^) mice during a10-minute observation period. Mice that received buffer alone, lowest concertation of FPH tested or amputations performed at 30 minutes vs 1 minute post-product administration had a significant increase in the fraction of tails bleeding as analyzed by log-rank test (*P* < 0.001). Statistical significance for thrombus area was determined by Mann-Whitney *U* test. ∗∗∗*P* < 0.001 relative to healthy donors; ∗∗∗*P* < 0.001 high relative to low concentration of FPH. ns = not significant.

FPH is known to range in size from 1 to 5 μm and possess a morphology more consistent with that of activated platelets including surface expression of PS.^22^^,^^23^ To determine whether this agent could support hemostasis as effectively as platelets isolated from healthy donors, we performed a standard tail tip amputation using NOD/SCID mice expressing the human VWF-A1 domain. Previously, we demonstrated that these animals have a bleeding diathesis due to the inability of GPIb alpha on murine platelets to support effective interactions with the A1 domain of human VWF in flow.^28^ In the absence of FPH, 86% of animals bled profusely throughout the duration of the experiment (10 minutes), consistent with previous studies. By contrast, restoration in hemostasis was achievable at the level observed for freshly isolated human platelets when FPH was administered at dosing concentrations of 4.4 × 10^9^ and 8.7 × 10^9^ particles/kg (Figure 2A) (*P* = 0.48 and *P* = 0.55, respectively, as compared with healthy donors). In a separate experiment to assess clot stability, hemostasis was maintained for up to 20 minutes with only 3 mice briefly rebleeding (*t* = 14 seconds, 33 seconds, and 207 seconds) (Figure 2B). Moreover, the ability of FPH to support the formation of a hemostatic plug was time restricted as 92% mice that underwent tail amputation 30 minutes after product administration continued to bleed (Figure 2C), results consistent with its short circulating lifetime.^34^ Of note, plasma VWF was essential to this process as FPH was unable to correct the severe bleeding diathesis associated with mice deficient in this plasma protein.

**Figure 3:**
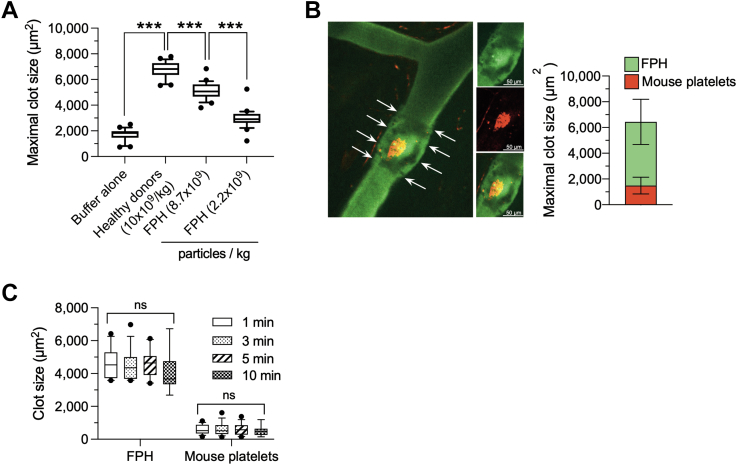
Accrual of FPH and Mouse Platelets at Sites of Arterial Injury Maximal area (μm^2^) occupied by CFDA-SE–labeled FPH at the indicated dosing concentrations after laser-induced arteriole injury in VWF^HA1^ mice (n = 10 mice for each group, 3 arteriole injuries per mouse; *t* = 2 minutes). Results are compared with healthy donor platelets or endogenous mouse platelets (buffer alone) in the absence of FPH. (B) Insert: Representative photomicrograph depicting maximal accumulation of CFDA-SE–labeled FPH (green), rhodamine-labeled mouse platelets (red), and composite image (yellow) at the site of arteriole injury. Arrows depict the area occupied by FPH within the region of arteriole injury. Composition of clot based on % of total maximal area occupied by FPH (green) vs mouse platelets (red) (n = 5 mice, 3 arterioles per mouse; *t* = 2 minutes). (C) Area (μm^2^) occupied by CFDA-SE–labeled FPH (8.7 × 10^9^ particles/kg) vs rhodamine 6G–labeled mouse platelets as determined by 2 channel confocal microscopy at 1, 3, 5, and 10 minutes post-injury. The central box represents the values between the 10th and 90th percentiles, and the middle line is the mean. Statistical significance for thrombus area was determined by either Mann-Whitney *U* test or analysis of variance for comparison of multiple groups. ∗∗∗*P* < 0.001 relative to healthy donors; ∗∗∗*P* < 0.001 high relative to low concentration of FPH. Abbreviations as in Figure 2.

Consistent with our hemostatic studies, circulating CFDA-SE–labeled FPH directly formed a physical barrier at sites of laser-induced arteriole injury that was essential for the cessation of bleeding (Figure 3A, Video S1). The area occupied by FPH was dose dependent with a mean ± SD of 5,109 ± 656 μm^2^ (95% CI: 4,832-5,368 μm^2^) and 2,955 ± 710.8 μm^2^ (95% CI: 2,655-3,255 μm^2^) for the higher vs lower concentration of product, respectively. To demonstrate that FPH accrual was not critically dependent on circulating platelets, we performed 2-channel confocal intravital microscopy after injecting labeled FPH into animals that received an intravenous bolus of rhodamine 6G to detect mouse platelets. Upon induction of arteriole injury, thrombi that formed were composed mainly of FPH (84.3% total thrombus area) with minimal contribution of murine platelets (Figure 3B). This is further supported by the lack of increased deposition of mouse platelets over an observation period of 10 minutes (Figure 3C) (*P* = 0.91).

### FPH and Effect of P2Y_12_ Receptor Antagonists on Hemostatic CLOT Formation

**Figure 4:**
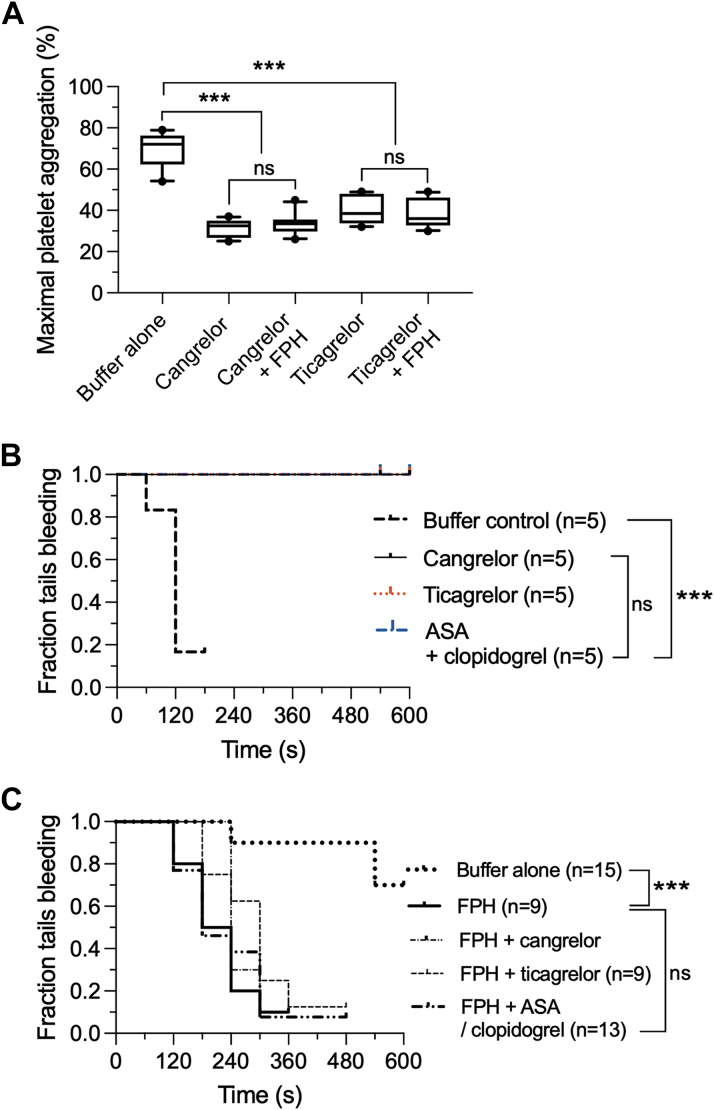
FPH Supports Hemostasis in the Presence of P2Y_12_ Receptor Antagonists (A) Determination of the ability of FPH to scavenge cangrelor or ticagrelor in plasma. This was accomplished by measuring maximal ADP-induced aggregation of platelets from healthy donors (n = 5) suspended in autologous platelet-poor plasma containing either cangrelor (0.1 μmol/L) or ticagrelor (2 μmol/L) that was first incubated with and then depleted of FPH (1 × 10^8^ particles/mL) before performing light transmission aggregometry (LTA). (B) Fraction of tails bleeding at 10 minutes for wild-type mice that received cangrelor, ticagrelor, or a combination of clopidogrel and acetylsalicylic acid (ASA) as compared with buffer control. (C) Fraction of tails bleeding at 10 minutes for VWF^HA1^ NOD/SCID mice that received cangrelor, ticagrelor, or a combination of clopidogrel and ASA before administering a hemostatic concentration of FPH (4.4 × 10^9^ particles/kg). The central box represents the values between the 10th and 90th percentiles, and the middle line is the mean. Statistical significance for LTA was determined by one-way analysis of variance for comparison of the effects of P2Y_12_ inhibitors to buffer alone (∗∗∗*P* < 0.001). A Mann-Whitney *U* test was used to determine significance between P2Y_12_ antagonist–treated plasma in the absence to presence of FPH. Mice that received the P2Y_12_ antagonists had no significant increase in fraction of tails bleeding at 10 minutes after administration of FPH compared with FPH alone and analyzed by log-rank test (*P* = 0.461). Abbreviations as in Figures 1 and 2.

Previously, it has been shown in a preclinical study that the antiplatelet effects of P2Y_12_ antagonists can be reversed by platelet-mimicking nanosponges that bind to and remove these agents from the blood.^35^ To determine whether our investigation product could also act as a sink for unbound P2Y_12_ receptor antagonists, platelets from healthy donors were suspended in autologous PPP that was first treated with either cangrelor or ticagrelor, then incubated with and subsequently depleted of FPH (1 × 10^8^ particles/mL) before performing LTA with ADP as agonist. Results indicate that platelet reactivity remained diminished and essentially unchanged despite incubation with FPH. Mean maximal platelet aggregation for cangrelor was 31.3% ± 4.2% (95% CI: 28.3%-34.3%) vs 33.6% ± 5.2% (95% CI: 29.9%-37.3%) in the absence or presence of FPH, respectively; results for ticagrelor were 39.8% ± 6.9% (95% CI: 34.9%-44.8%) vs 38.8% ± 7.1% (95% CI: 33.7%-43.9%), respectively (Figure 4A).

We next evaluated the ability of P2Y_12_ inhibitors to directly affect the hemostatic property of FPH. To this end, we first confirmed the ability of cangrelor, ticagrelor, or a combination of ASA and clopidogrel to significantly impair hemostasis in mice expressing the native (WT) murine A1 domain of VWF (Figure 4B) (*P* < 0.001 compared with buffer alone).^27^^,^^36^^,^^37^

Using the identical concentrations and dosing regimen but this time in VWF^HA1^ animals, none of these antiplatelet agents impaired the ability of FPH to support effective hemostasis as evidenced by the nearly identical tail bleeding times in the absence or presence of antagonists (Figure 4C) (*P* = 0.46).

### FPH Augments the Hemostatic Capacity of DAPT-Exposed Platelets

**Figure 5:**
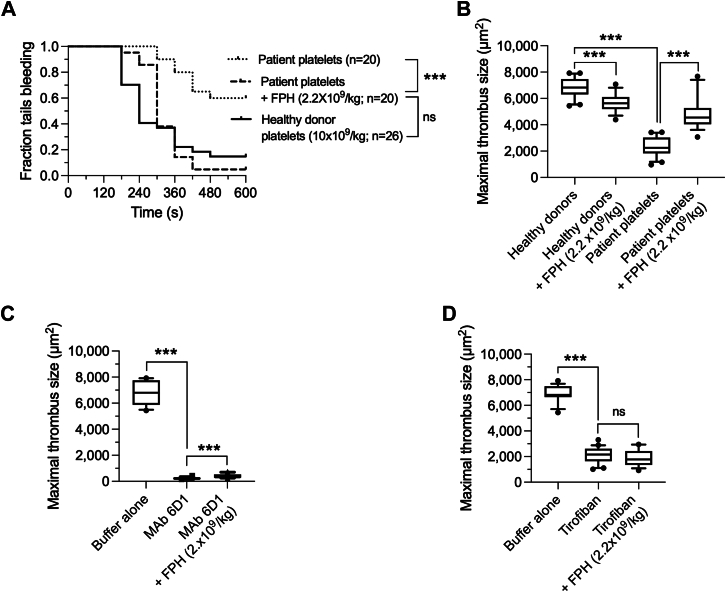
FPH Augments the Hemostatic Properties of DAPT-Exposed Platelets (A) Fraction of tails bleeding at 10 minutes for VWF^HA1^ mice after intravenous administration of platelets (10 × 10^9^/kg) from healthy donors or patients on DAPT. FPH was infused 1 minute after platelets at a concentration that does not independently support hemostasis (2.2 × 10^9^ particles/kg). (B) Maximal area (μm^2^) occupied by calcein-labeled platelets from the same individuals before and after administration of FPH at an identical concentration (n = 10 mice per group, 10 individuals for each of the 2 groups, 3 arteriole injuries per mouse). (C) Maximal area (μm^2^) occupied by calcein-labeled platelets from healthy donors in the presence of the GPIbα function blocking monoclonal antibodies (mAb) 6D1 (50 μg) or (D) αIIb/*β3 inhibitor* tirofiban (25 μg/kg body weight bolus followed by 0.15 μg/kg/min continuous infusion) in the absence or presence of FPH (n = 5 mice per condition, minimum 3 arteriole injuries per mouse). The central box represents the values between the 10th and 90th percentiles, and the middle line is the mean. Statistical significance for thrombus area was determined by Mann-Whitney *U* test. ∗∗∗*P* < 0.001 relative to healthy donors or study patient platelets in the absence of FPH. Mice infused with patient platelets had significant reduction in fraction of tails bleeding at 10 minutes after administration of FPH as analyzed by log-rank test (∗∗∗*P* < 0.001). Abbreviations as in Figures 1 and 2.

After demonstrating that P2Y_12_ antagonists do not impair the hemostatic properties of FPH, we next determined whether this investigation product could enhance the ability of patient platelets to form an effective hemostatic plug in vivo. To this end, FPH was administered at a concentration (2.2 × 10^9^ particles/kg) that by itself is incapable of preventing bleeding in VWF^HA1^ mice. In its absence, 60% of mice that received human platelets exposed to DAPT bled profusely throughout the duration of the experiment (10 minutes) (Figure 5A) (*P* < 0.001 as compared with healthy donors). Subsequent administration of FPH, however, enhanced the hemostatic properties of these dysfunctional platelets as evidenced by the significant reduction in percentage of animals that continued to bleed by the end of the observation period (5%; *P* < 0.001 compared with no FPH).

To directly demonstrate that FPH could augment the accrual of DAPT exposed platelets at sites of vascular damage, VWF^HA1^ mice that received patient platelets underwent laser-induced arterial injury before and after an infusion of a nonhemostatic concentration of the investigational product (2.2 × 10^9^ particles/kg). As aforementioned, thrombi that formed were significant smaller than those from healthy donors in the absence of FPH (Figure 1E). Of note, subsequent administration of the investigation product resulted in an ∼2-fold increase in clot size for the former (Figure 5B, Video S2). By contrast, FPH did not augment the thrombotic properties of platelets from healthy donors or mice possessing the native (WT) form of murine VWF (Figure 5B, Supplemental Figure S1). In fact, the opposite was true as there was a modest, but significant, reduction in maximal clot size formed in the presence of platelets from healthy donors with a mean area of 5,679 ± 698.4 μm^2^ (95% CI: 5,292-6,066 μm^2^; *P* < 0.001). Of note, there was no apparent perturbation in coagulation parameters, namely PT and aPTT values, post-FPH administration (Supplemental Figure S2).^38^ In addition, the platelet receptor GPIb alpha and integrin αIIbβ3 were also essential to this process as function blockade with either mAb 6D1 or tirofiban, respectively, limited FPH induced augmentation in platelet accrual (Figures 5C and 5D).

### FPH Promotes Thrombin-Mediated Accrual of DAPT-Exposed Platelets

**Figure 6:**
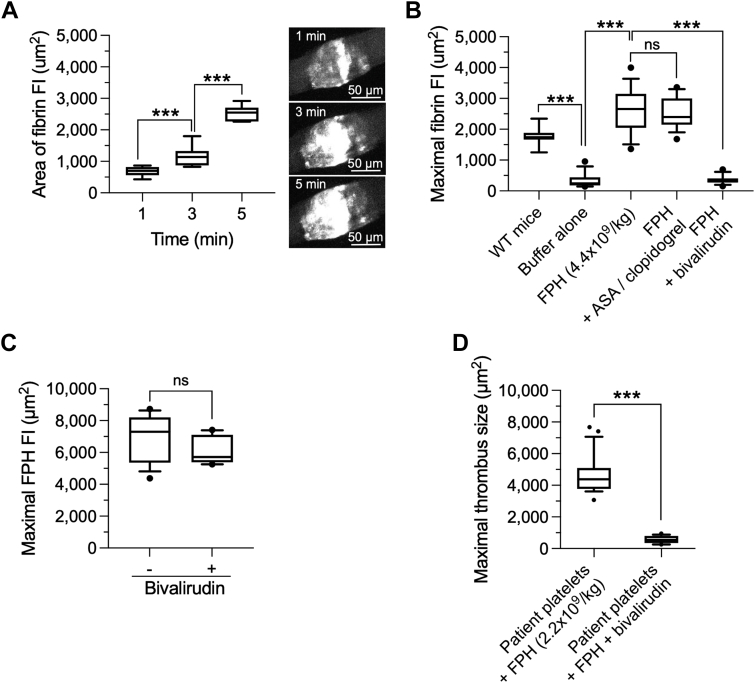
FPH-Induced Thrombin Generation and Contribution to Clot Formation (A) Time course depicting the deposition of Alexa Fluor 647–conjugated human fibrin in laser-injured arterioles of VWF^HA1^ mice. Total fibrin area was determined by flattening data sets along the z-axis as maximum intensity projections. Insert: Representative photomicrographs depicting fibrin accumulation at the designated time points. (B) Area (μm^2^) of fibrin deposited in arterioles of VWF^HA1^ mice at 5 minutes post injury. Animals were gavaged with ASA and clopidogrel or received an intravenous bolus of bivalirudin before an injection of Alexa Fluor 647–conjugated human fibrinogen. This was followed by buffer control or a hemostatic concentration of FPH (n = 5; minimum 3 arterioles per mouse). (C) Maximal area (μm^2^) occupied by CFDA-SE–labeled FPH in the absence or presence of bivalirudin (n = 5; minimum of 3 arterioles per animal). (D) Maximal thrombus size (μm^2^) for calcein-labeled platelets from patients on DAPT in the absence or presence of a concentration of FPH that alone cannot support hemostasis (±bivalirudin). The central box represents the values between the 10th and 90th percentiles, and the middle line is the mean. Statistical significance was determined using Mann-Whitney *U* test. ∗∗∗*P* < 0.001. WT = wild-type; other abbreviations as in Figures 1, 2, and 4.

Although FPH has the capacity to restore the ability of platelets from patients on DAPT to form stable hemostatic plugs, the mechanism(s) by which this occurs in vivo is unknown. As exposed platelet membrane *PS* can contribute to the production of thrombin by serving as a procoagulant surface, we speculated that the investigational product may induce the generation of this potent platelet agonist at sites of vascular injury thereby providing an alternative pathway for activation. To this end, VWF^HA1^ mice were injected with Alexa Fluor 647–conjugated human fibrinogen and then administered buffer control or CFDA-SE–labeled FPH at a concentration that supports hemostasis (4.4 × 10^9^ particles/kg). As thrombin converts fibrinogen to fibrin, the total area of accumulation of the latter was determined over a period of 5 minutes post-vascular injury. This was accomplished by generating maximum intensity projections of images taken along the z-axis that encompassed the entire area of vascular damage. Compared with buffer control, the infusion of FPH resulted in a time-dependent augmentation in fibrin deposition as evidenced by the increase in mean maximal area of fluorescence from 682 ± 160.5 μm^2^ (95% CI: 533.3-830.4 μm^2^) at 1 minutes post-injury to 1,171 ± 328.3 μm^2^ (95% CI: 867.8-1,474 μm^2^) and 2,515 ± 244.6 μm^2^ (95% CI: 2,289-2,742 μm^2^) at 3 minutes and 5 minutes, respectively (Figure 6A). Evidence that mouse platelets play a limited role in participating in this process is shown by the 5-fold reduction in fibrin deposition that occurred at sites of arteriole injury in VWF^HA1^ mice (buffer alone) as compared with WT control animals (Figure 6B) (*P* < 0.001). Moreover, FPH-induced fibrin deposition was unaffected by pretreatment of VWF^HA1^ mice with ASA and clopidogrel: mean fibrin area of 2,651 ± 690.3 μm^2^ (95% CI: 2,183-2,998 μm^2^) and 2,512 ± 472.4 μm^2^ (95% CI: 2,225-2,743 μm^2^; *P* = 0.46 as compared with FPH alone). Importantly, intravenous infusion of the direct thrombin inhibitor bivalirudin resulted in an ∼8-fold reduction in fibrin deposition in the presence of the identical dosing concentration of FPH. Of note, thrombin inhibition did not affect the accumulation of FPH at sites of vascular injury (Figure 6C) (*P* = 0.20). It did, however, limit the ability of the investigational product to augment the accrual of platelets isolated from patients on DAPT (Figure 6D, Video S3). Notably, the total area occupied by human platelets was reduced by >80% in the presence of bivalirudin with mean ± SD fibrin area of 4,648 ± 1,177 μm^2^ (95% CI: 4,162-5,134 μm^2^) and 564 ± 233.1 μm^2^ (95% CI: 448-679.9 μm^2^; *P* ≤ 0.001 as compared with absence of bivalirudin).

### Thrombin Inhibition or PS Blockade Limits FPH Mediated Hemostasis

**Figure 7:**
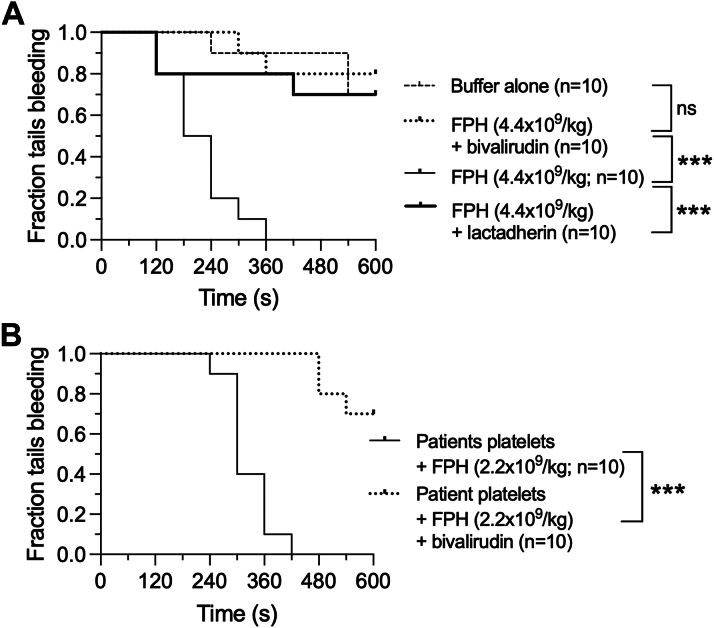
Contribution of FPH-Induced Thrombin Generation to Hemostasis (A) Fraction of tails bleeding at 10 minutes for VWF^HA1^ mice that received a hemostatic concentration of FPH (4.4 × 10^9^ particles/kg) followed by the administration of either buffer control or bivalirudin. FPH was also preincubated with lactadherin (150 μg/mL) 30 minutes before injection to assess the contribution of exposed phosphatidylserine to the hemostatic process. (B) Fraction of tails bleeding for VWF^HA1^ mice that received platelets from patients on DAPT followed by the administration of a non-hemostatic concentration of FPH and either buffer control or bivalirudin. Mice that received FPH alone or in combination with platelets from study patients had a significant increase in the fraction of tails bleeding at 10 minutes in the presence of bivalirudin or lactadherin as analyzed by log-rank test (∗∗∗*P* < 0.001). Abbreviations as in Figures 1 and 2.

To assess the importance of FPH-induced thrombin generation in supporting the formation of an effective hemostatic plug, we first determined whether bivalirudin could impair the ability of this investigational product to prevent bleeding in VWF^HA1^ mice. Consistent with the reduction in fibrin deposition at sites laser-induced injury, administration this thrombin antagonist after infusion of a hemostatic dose of FPH (4.4 × 10^9^/kg) resulted in 80% increase in the number of animals that continued to bleed at 10 minutes (Figure 7A) (*P* < 0.001). Similarly, preincubation of FPH with a concentration of lactadherin that blocks PS participation in coagulation limited thrombin generation ex vivo and significantly impaired hemostasis as evidenced by >75% of mice continuing to bleed at 10 minutes (Figure 7A, Supplemental Figure S3) (*P* < 0.001). Administration of bivalirudin also reversed the ability of the investigational product to bypass the effects of DAPT (Figure 7B) (*P* < 0.001 compared with FPH alone).

## Discussion

The present study provides detailed insight into the potential value of a platelet derived product that is characterized by ease of storage, immediate availability, extended shelf life of 3 years, and ability to use in any recipient (leukoreduced, Blood Group O). These are desirable attributes as reduced platelet donations (exaggerated by the Covid pandemic) and a limited shelf-life of 7 days has led to a critical shortage of this blood product.^39^ In support of the clinical use of FPH is our in vivo observations demonstrating that it not only forms an effective physical barrier at sites of arterial injury but can also augment the deposition and hemostatic properties of hyporeactive platelets isolated from patients on DAPT. Of note, this was not achievable in animals deficient in VWF, suggesting that the presence of this multimeric plasma protein, or at least the human A1 domain, is required for optimal activity. This is further supported by the inability of FPH to augment the accumulation of platelets in which GPIb alpha interaction with VWF-A1 was blocked with mAb 6D1.

Clearly, FPH can work independently of circulating platelets, but do they possess additional attributes that would be of benefit in the clinical management of bleeding in patients on DAPT? Previously, it has been shown that a perfluorocarbon-based particle expressing a recombinant P2Y_12_ receptor can serve as a sink for ticagrelor, a reversible inhibitor of the ADP receptor on platelets.^35^ This was not the case for FPH as evidenced by the inability to prevent P2Y_12_ receptor antagonism of healthy donor platelets when incubated with autologous plasma containing either cangrelor or ticagrelor. Despite this finding, these inhibitors, as well as the combination of ASA and clopidogrel, did not affect the ability of FPH to correct the bleeding diathesis in VWF^HA1^ mice. This suggests a mechanism of action distinct from that reported for platelet-mimicking nanosponges as FHP directly bypassed the inhibitory effects of these antagonists, resulting in enhancement in the hemostatic properties of DAPT-exposed platelets, even at a concentration that itself did not prevent bleeding. As aforementioned, the investigational product did not augment the accumulation of platelets from healthy donors or in WT mice nor result in the formation of occlusive thrombi even at the highest concentration tested); moreover, there were no significant changes in PT/aPTT after FPH administration. This is an important observation as the safety/efficacy goal of any potential therapy is to administer the lowest dose that achieves the desired outcome.

How does FPH counteract the antihemostatic effects associated with the administration of aspirin and clopidogrel to patients? The platelet surface plays a central role in the promotion and regulation of thrombin generation by interacting with coagulation factors. This is due in part to exposure of the anionic aminophospholipid PS on the activated platelet surface that facilitates assembly of the intrinsic tenase and prothrombinase complexes, thereby accelerating local thrombin generation.^40^^,^^41^ FPH are known to exist in activated state as evidenced by surface expression of P-selectin, PS, and αIIb β3.^26^ Indeed, we demonstrate that this investigational product not only generates thrombin in vivo as evidenced by the deposition of fibrin, but can do so even in the presence of ASA and clopidogrel. In this context, FPH can serve as a connector between primary and secondary hemostasis due to the ability to rapidly accumulate at sites of arterial injury and promote thrombin-mediated fibrin deposition and clot stability. The latter was confirmed by the near abrogation of fibrin and DAPT-exposed platelets at sites of arterial injury in the presence of bivalirudin and in the impairment in hemostasis that occurs after preincubation of FPH with lactadherin to block PS-mediated thrombin production.

Another unique aspect of this study was the ability to rapidly assess the degree of DAPT-induced platelet inhibition in a highly, multiplexed manner using <1 mL of whole blood. This was achievable through microfluidic technology that closely simulates conditions at sites of vascular injury. This is of importance as the in vivo efficacy of pharmacological agents such as aspirin and clopidogrel is determined by multiple factors that cannot be measured by LTA such as the degree of surface-mediated platelet activation and convective removal of autocrine agonists from the injury site.^40^ Of the 27 patients enrolled in this preclinical trial, all had significant reduction in ADP-induced platelet aggregation as measured by LTA. Importantly, results were consistent with the observed reduction in thrombus size at sites of arterial injury in VWF^HA1^ mice validating the ability of the device to simulate in vivo conditions.

Safety is an important aspect of any therapy directed at reversing or overcoming the effects of antiplatelet agents. Importantly, 2 phase I trials have been conducted with no adverse events detected in participants upon infusion of FPH or during follow-up monitoring.^25^^,^^26^ As aforementioned, our data suggest that should thrombosis be suspected in response to treatment, a thrombin inhibitor such as heparin could prevent excessive clot formation. Overall, this study provides the first in vivo evidence that FPH can augment the hemostatic properties of platelets from patients on DAPT, warranting further evaluation for use in the treatment of severe bleeding in this patient population.

### Study Limitations

A clinical trial will ultimately be required to determine the safety and efficacy of FPH in managing acute, severe bleeding in patients with cardiovascular disease on DAPT.

## Conclusions

Major bleeding events remain a major complication of antiplatelet therapy in patients with cardiovascular disease and can necessitate urgent restoration in hemostasis. However, limited options exist for reversing the effects of such therapies, which can be compounded by a suboptimal response to platelet transfusion due to direct inhibition by APA. By enrolling patients with significant coronary artery disease on DAPT and utilizing a humanized animal model of vascular injury, we demonstrate that FPH has potential value as a therapy to bypass P2Y_12_ inhibition and restore the hemostatic properties of dysfunctional platelets in vivo. Moreover, the activity of FPH can be modulated to favor hemostasis over vessel thrombosis by limiting the concentration infused so that they work in concert with DAPT-exposed platelets. Current data also suggest that if faced with a suspected adverse reaction such as thrombosis, a thrombin inhibitor would limit excessive clot formation by reducing the production of this potent agonist by surface-adherent FPH. Overall, this study demonstrated the potential in vivo efficacy of FPH offering a titratable and rapidly reversible strategy for the management of bleeding complications induced by antiplatelet therapy.

Perspectives **COMPETENCY IN MEDICAL KNOWLEDGE:** DAPT consisting of aspirin and a P2Y_12_ receptor antagonist reduces the risk of ischemic events post-stent placement but at the cost of increased risk of bleeding. Although a feared complication that may lead to significant morbidity and mortality, DAPT-associated bleeding represents a clinical challenge in a field where limited definitive evidence exists regarding best management. That said, acute severe hemorrhage whether due to a spontaneous gastrointestinal bleed, trauma, or urgent surgical procedure would require immediate restoration in hemostasis. Platelet transfusions are often used in emergency situations, but they may not be effective if active drug is still present. FPH provides a potential alternative therapy as the investigational product targets sites of arterial injury, augments accumulation and potentiates the reactivity of DAPT exposed platelets through thrombin generation even in the presence of aspirin and P2Y_12_ antagonists.

**TRANSLATIONAL OUTLOOK:** From a safety perspective, FPH did not appear to amplify the thrombotic properties of platelets from healthy individuals and its activity could be modulated by a thrombin inhibitor. Based on these observations and initial safety profile in phase 1 clinical trials involving healthy adult volunteers and thrombocytopenic patients with hematological malignancies, it is reasonable to further assess safety and efficacy in cardiovascular patients on DAPT requiring emergent restoration in hemostasis.

## Funding Support and Author Disclosures

This study was sponsored by Cellphire, Inc., and the New York Fund for Innovation in Research and Scientific Talent. Dr Moskowitz is employed by Cellphire, Inc., whose product was studied in the present work. All other authors have reported that they have no relationships relevant to the contents of this paper to disclose.

## Appendix

Video 1 FPH Accrue at Sites of Arterial Injury and form a Hemostatic Plug Video 1 (left) demonstrates the lack of interaction between CFDA-SE labeled FPH and intact vascular endothelium in the cremaster muscle of a VWF HA1 mouse. Video 2 (right) demonstrates the ability FPH to form an effective hemostatic plug, preventing further escape of blood from a laser-injured arteriole in the same animal.

Video 2 FPH Augment the Accumulation of DAPT-Exposed Platelets From Patients at Sites of Vascular Injury Video 1 (top) demonstrates the inability of platelets from patients on aspirin and clopidogrel to form stable thrombi at sites of arterial injury. Video 2 (bottom) demonstrates the augmentation in platelet accumulation after administration of FPH in a laser-injured arteriole in the same animal.

Video 3 Bivalirudin Reverses FPH-Induced Accumulation of DAPT-Exposed Platelets From Patients Video 1 (left) demonstrates the augmentation in platelet accumulation after administration of FPH. Video 2 (right) demonstrates the inability of FPH to augment platelet accumulation after administration of bivalirudin in the same animal.
