# Supplementary material for: Preclinical Efficacy of a Hemostatic Agent in Overcoming Dual Antiplatelet Therapy
Source: JACC Basic Transl Sci. 2025 Aug 15;10(9):101356. doi: 10.1016/j.jacbts.2025.101356 (PMC12390928; doi:10.1016/j.jacbts.2025.101356)
Supplement: Supplemental Material [file mmc4.docx]

**Supplementary Materials for**

**Preclinical Efficacy of a Hemostatic Agent in Overcoming Dual Antiplatelet Therapy**

Evgeni Efimenko, PhD, Hairu Zhao, MS, Keith Moskowitz, PhD, Conrad Smith, MD, Robert Pyo, MD, Thomas G. Diacovo, MD

**This PDF file includes**

Figure S1- S3

Video Legends S1 – S3

ns

**Fig. S1.** Maximal thrombus area (µm^2^) occupied by circulating, rhodamine 6G labeled platelets in WT mice after intravenous administration of buffer control or FPH (2.2x10^9^ particles/kg; n=5 mice per group, 3 arteriole injuries per mouse). The central box represents the values between the 10th and 90th percentiles, and the middle line is the mean. Statistical significance was determined using Mann-Whitney U test.

**A**

**B**

**Fig. S2.** Coagulation parameters in platelet-poor plasma. (A) PT, prothrombin time (n=3); (B) aPTT, activated partial thromboplastin time (n=3) in VWF ^HA1^ mice 30 min after receiving an infusion of buffer or FPH (4.4 x 10^9^ particles /kg). Each dot represents one animal. Data represent the mean ± SEM. Statistical significance was determined using Mann-Whitney U test.

**Fig. S3.** Lactadherin blocking of FPH-induced thrombin generation. Dose response curve (simple linear regression) of FPH-induced thrombin generation with escalating concentrations of lactadherin to block exposed phosphatidylserine.

**Video S1. FPH accrue at sites of arterial injury and form a hemostatic plug**

Video 1 (left) demonstrates the lack of interaction between CFDA-SE labeled FPH and intact vascular endothelium in the cremaster muscle of a VWF ^HA1^ mouse. Video 2 (right) demonstrates the ability FPH to form an effective hemostatic plug, preventing further escape of blood from a laser-injured arteriole in the same animal.

**Video S2. FPH Augment the Accumulation of DAPT-exposed Patient Platelets at Sites of Vascular Injury**

Video 1 (top) demonstrates the inability of platelets from patients on aspirin and clopidogrel to form stable thrombi at sites of arterial injury. Video 2 (bottom) demonstrates the augmentation in platelet accumulation after administration of FPH (2.2x10^9^ particles/kg) in a laser-injured arteriole in the same animal.

**Video S3. Bivalirudin reverses FPH-induced accumulation of DAPT-exposed Patient Platelets**

Video 1 (left) demonstrates the augmentation in platelet accumulation after administration of FPH (2.2x10^9^ particles/kg). Video 2 (right) demonstrates the inability of FPH to augment platelet accumulation after administration of bivalirudin in the same animal.
